# Overuse and underuse of thromboprophylaxis in medical inpatients

**DOI:** 10.1016/j.rpth.2023.102184

**Published:** 2023-08-23

**Authors:** Barbara Kocher, Pauline Darbellay Farhoumand, Damiana Pulver, Basil Kopp, Damien Choffat, Tobias Tritschler, Peter Vollenweider, Jean-Luc Reny, Nicolas Rodondi, Drahomir Aujesky, Marie Méan, Christine Baumgartner

**Affiliations:** 1 Department of General Internal Medicine, Inselspital, Bern University Hospital, University of Bern, Bern, Switzerland; 2 Division of General Internal Medicine, Department of Medicine, Geneva University Hospitals (HUG), Geneva, Switzerland; 3 Division of Internal Medicine, Department of Medicine, Lausanne University Hospital (CHUV), Lausanne, Switzerland; 4 Ottawa Hospital Research Institute, University of Ottawa, Ottawa, Ontario, Canada; 5 Institute of Primary Health Care (BIHAM), University of Bern, Bern, Switzerland

**Keywords:** hospitalization, prescriptions, prevention, prophylaxis, risk assessment, venous thromboembolism, venous thrombosis

## Abstract

**Background:**

Thromboprophylaxis (TPX) prescription is recommended in medical inpatients categorized as high risk of venous thromboembolism (VTE) by validated risk assessment models (RAMs), but how various RAMs differ in categorizing patients in risk groups, and whether the choice of RAM influences estimates of appropriate TPX use is unknown.

**Objectives:**

To determine the proportion of medical inpatients categorized as high or low risk according to validated RAMs, and to investigate the appropriateness of TPX prescription.

**Methods:**

This is a prospective cohort study of acutely ill medical inpatients from 3 Swiss university hospitals. Participants were categorized as high or low risk of VTE by validated RAMs (ie, the Padua, the *International Medical Prevention Registry on Venous Thromboembolism*, simplified, and original Geneva scores). We assessed prescription of any TPX at baseline. We considered TPX prescription in high-risk and no TPX prescription in low-risk patients as appropriate.

**Results:**

Among 1352 medical inpatients, the proportion categorized as high risk ranged from 29.8% with the *International Medical Prevention Registry on Venous Thromboembolism* score to 66.1% with the original Geneva score. Overall, 24.6% were consistently categorized as high risk, and 26.3% as low risk by all 4 RAMs. Depending on the RAM used, TPX prescription was appropriate in 58.7% to 63.3% of high-risk (ie, 36.7%-41.3% underuse) and 52.4% to 62.8% of low-risk patients (ie, 37.2%-47.6% overuse).

**Conclusion:**

The proportion of medical inpatients considered as high or low VTE risk varied widely according to different RAMs. Only half of patients were consistently categorized in the same risk group by all RAMs. While TPX remains underused in high-risk patients, overuse in low-risk patients is even more pronounced.

## Essentials

• Risk models aim to identify medical inpatients at risk of venous thromboembolism.
• We assessed thromboprophylaxis prescribing using validated models to predict venous thromboembolism risk.
• Depending on the risk model, the proportion categorized as high risk varied from 30% to 66%.
• TPX is underused in 37% to 41% of high-risk patients and overused in 37% to 48% of low-risk patients.

## 1 Introduction

Venous thromboembolism (VTE), defined as deep vein thrombosis (DVT) or pulmonary embolism (PE), is a common complication of a hospitalization. About 50% of all VTE events occur during or up to 3 months after hospitalization (ie, hospital-acquired VTE) [[1], [2], [3]]. VTE risk is particularly high after surgery [4], but hospitalization for acute medical illness is also a risk factor [1]. Up to 75% of all hospital-acquired VTE events occur in non-surgical patients [5]. VTE is associated with high mortality and morbidity and the consequences of VTE, especially in case of PE, can be fatal [1,6]. Randomized controlled trials performed 2 decades ago have shown that pharmacologic thromboprophylaxis (TPX) in medical inpatients was effective in reducing the VTE risk [[7], [8], [9]]. Based on available evidence, clinical guidelines recommend administering pharmacologic TPX with low-molecular-weight heparin (LMWH) or fondaparinux in a prophylactic dose to medical inpatients at increased VTE risk during their inpatient stay, provided there is no active bleeding and no increased risk of major bleeding [10]. While in surgical inpatients the VTE risk is determined by the type and duration of intervention [4], risk assessment in medical inpatients is more difficult and requires consideration of multiple factors [11,12].

To target the use of pharmacologic TPX and to simplify VTE risk stratification in medical inpatients, guidelines suggest the use of validated risk assessment models (RAMs),^10^ such as the Padua [13], the *International Medical Prevention Registry on Venous Thromboembolism* (IMPROVE) [14,15], the original [16,17], or simplified [18] Geneva score. These RAMs provide a summary score based on differently weighted VTE risk factors. Depending on the summary score, patients are categorized into either a low- or high-VTE risk group, with the aim to guide provision of TPX to those at high risk [[13], [14], [15], [16], [17], [18]]. Despite existing guidelines, pharmacologic TPX is often inappropriately used in this population. Previous studies have reported that the proportion of high-risk patients with an appropriate prescription of TPX is only 40%, whereas almost half of all low-risk patients are prescribed unnecessary TPX [12,17], although the definition of appropriate and inappropriate prescription of TPX varies widely depending on the criteria used [19]. The comparative performance of various RAMs to predict VTE has been studied [18], although it is unclear how they differ categorizing patients in high and low VTE risk groups. In addition, how the choice of a particular RAM influences estimates of overuse and underuse of TPX is currently unknown.

The aim of this study is to determine the proportion of medical inpatients categorized as high or low risk of VTE according to validated RAMs, and to investigate the appropriateness of TPX in high-risk and low-risk patients based on each RAM, using data from a prospective cohort study of medical inpatients.

## 2 Methods

### 2.1 Setting and population

We used data from the *Risk Stratification for Hospital-Acquired Venous Thromboembolism in Medical Patients* (RISE) study, a multicenter, non-interventional prospective cohort study of adult patients hospitalized for acute illness in general internal medicine wards of 3 Swiss university hospitals between May 2020 and January 2022.

The trial protocol has been previously published [20]. On weekdays, study personnel screened consecutive patients on general internal medicine wards that were newly admitted to the hospital. Inclusion criteria were age ≥18 years and admission for hospitalization >24 hours to general internal medicine due to an acute illness. Exclusion criteria were the need for therapeutic anticoagulation (eg, atrial fibrillation), life expectancy <30 days, insufficient proficiency of the German or French language, unwillingness to provide informed consent, and prior enrolment in the study. Patients who were unable to give informed consent (eg, due to mental illness or cognitive impairment) were not excluded from participation, because the risks of VTE, immobilization, and associated adverse outcomes are particularly high in the elderly [21,22] in whom cognitive impairment is more prevalent. Written informed consent was obtained from their legally authorized representative. Eligible study participants were enrolled within 72 hours of admission. The study was approved by the Ethics committees of the participating sites.

### 2.2 Baseline data collection

Trained study personnel collected baseline information about demographic characteristics, all items of selected validated RAMs (Padua [13], IMPROVE [14,15], original [16,17], and simplified [18] Geneva score; Table 1), other VTE risk factors, comorbidities, potential contraindications to pharmacologic TPX, and medications at admission with a potential antithrombotic effect. At the discharge visit, information about treatments during the current hospital stay was collected. Data were collected at the bedside and from electronic health records using standardized forms. Previous VTE was defined as prior DVT or PE. Hypercoagulable state or thrombophilia included diagnoses of antithrombin deficiency, activated protein C resistance, protein C or protein S deficiency, factor V Leiden, G20210A prothrombin-mutation, or antiphospholipid syndrome. Active cancer was defined as metastatic cancer, cancer treated with radiotherapy, chemotherapy, immunotherapy, or surgery within the past 6 months. Myeloproliferative syndrome referred to essential thrombocytopenia, polycythemia vera, myelofibrosis, or chronic myeloid leukemia. Cardiac failure was defined as diagnosis of acute or chronic heart failure with preserved or reduced ejection fraction in medical records, or a documented left ventricular ejection fraction of <40%. Respiratory failure was defined as an acute or chronic need for supplemental oxygen. Reduced mobility or immobilization was defined as anticipated bed rest with or without bathroom privileges for ≥3 days for the Padua score [13], as confinement to chair or bed with or without bathroom privileges for ≥7 days immediately prior to and during hospital admission for the IMPROVE score [14,15], and as complete bedrest or inability to walk for 30 minutes per day during ≥3 days for the original [16,17] and simplified [18] Geneva score. Obesity referred to a body mass index of ≥30 kg/m^2^. Hormonal treatment referred to hormonal contraception, postmenopausal hormone therapy, or antitumor therapy containing estrogen, ethinylestradione, or estradiol. Contraindications to pharmacologic TPX included liver failure and any other active bleeding disorders, active bleeding, or hemorrhagic transformation of acute ischemic stroke [12]. Liver failure was defined as diagnosis of liver failure in medical records, or cirrhosis with spontaneous international normalized ratio >2. Active bleeding disorder referred to the presence of any bleeding disorder except for liver disease, eg, hemophilia, von Willebrand disease, idiopathic thrombocytopenia. For each participant, the Padua, the IMPROVE, and the original and simplified Geneva score were calculated for the purpose of this study, as previously described **(**Table 1**)** [[13], [14], [15], [16], [17], [18]]. The treating physicians were not informed about the RAM scores, and none of the centers had a specific RAM integrated in their order sets or in their electronic medical records. However, all 3 hospitals had internal guidelines regarding the prescription of TPX. At the university hospitals in Bern and Lausanne, the Padua score was recommended to assess the indication for TPX prescription, while it was the simplified Geneva score at the university hospital of Geneva. While these internal guidelines indicated that non-pharmacologic TPX prophylaxis should be used in patients with both an increased bleeding and VTE risk, none of the guidelines explicitly listed bleeding risk factors or recommended the use of a formal bleeding risk score.

**Table 1:** RAMs for risk stratification of VTE in medical inpatients.

Score items	Points			
	Padua score [13]	IMPROVE score [14,15]	Original Geneva score [16,17]	Simplified Geneva score [18]
Previous VTE^a^	3	3	2	3
Hypercoagulable state or thrombophilia^b^	3	2	2	2
Active cancer^c^	3	2	2	2
Myeloproliferative syndrome^d^	-	-	2	
Cardiac failure^e^	1	-	2	2
Respiratory failure^f^		-	2	
Acute infection	1	-	2	2
Acute rheumatologic disorder		-	2	
Reduced mobility or immobilization^g^	3	1	1	2
Lower limb paralysis or paresis	-	2	-	-
Age >60 y	-	1	1	1
Age ≥70 y	1	-	-	-
Obesity or BMI ≥30 kg/m^2^	1	-	1	1
Recent stroke (≤3 mo)	1	-	2	1
Recent myocardial infarction (≤1 mo)		-	2	
Nephrotic syndrome	-	-	2	-
Hormonal treatment^h^	1	-	1	-
Recent travel >6 h (≤7 d)	-	-	1	-
Chronic venous insufficiency	-	-	1	-
Pregnancy	-	-	1	-
Dehydration	-	-	1	-
Recent trauma or surgery (<1 mo)	2	-	-	-
Stay in intensive or coronary care unit	-	1	-	-
**Cutoffs** [10,13,14,17,18]				
Low VTE risk	0-3	0-1	0-2	0-2
High VTE risk	≥4	≥2	≥3	≥3

BMI, body mass index; IMPROVE, *International Medical Prevention Registry on Venous Thromboembolism*; RAMs, risk assessment models; VTE, venous thromboembolism.

a Defined as prior deep vein thrombosis or pulmonary embolism.

b Defined as antithrombin deficiency, activated protein C resistance, protein C or protein S deficiency, factor (F)V Leiden, G20210A prothrombin-mutation, or antiphospholipid syndrome.

c Defined as metastatic cancer, or cancer treated with radiotherapy, chemotherapy, immunotherapy, or cancer surgery within last 6 months.

d Refers to essential thrombocytopenia, polycythemia vera, myelofibrosis, or chronic myeloid leukemia.

e Acute or chronic cardiac failure, defined as diagnosis of heart failure with preserved or reduced ejection fraction in medical records, or known left ventricular ejection fraction <40%.

f Acute or chronic respiratory failure, defined as need for supplemental oxygen.

g Defined as reduced mobility with anticipated bed rest with or without bathroom privileges for ≥3 days for the Padua score; defined as immobilization with confinement to chair or bed with or without bathroom privileges for ≥7 days immediately prior to and during hospital admission for the IMPROVE score; or defined as immobilization with complete bedrest or inability to walk for 30 minutes per day or ≥3 days for the original and simplified Geneva score.

h Refers to hormonal contraception, postmenopausal hormone therapy, antitumor therapy containing estrogen, ethinylestradione, or estradiol.

### 2.3 Outcomes

The primary outcome of the present analysis was the proportion of medical inpatients categorized as high or low risk of VTE by each RAM. Patients were categorized as high or low VTE risk according to each RAM at baseline; high VTE risk was defined as a score of ≥4 points on the Padua[13], ≥2 points on the IMPROVE [14,15], and ≥3 points on the original [16,17] and simplified [18] Geneva score **(**Table 1**)**.

Secondary outcomes were the prescription of any TPX, as well as underuse and overuse of TPX. Prescription of any TPX was defined as pharmacologic or mechanical TPX for at least one day, at baseline (ie, within 72 hours of admission) and anytime during the entire hospital stay. LMWH, unfractionated heparin (UFH), fondaparinux, or direct oral anticoagulants (apixaban, rivaroxaban) in a prophylactic dose were considered as pharmacologic TPX. Mechanical TPX was defined as use of lower extremity compression stockings or bandages, or intermittent pneumatic compression devices. Prescription of TPX was collected from medical records. We defined underuse of TPX as failure to prescribe TPX to patients categorized as high VTE risk, and overuse as prescription of TPX to patients categorized as low VTE risk based on a particular RAM. In other words, we considered TPX prescription in high-risk patients and no TPX prescription in low-risk patients as appropriate, in line with the *American College of Chest Physicians Evidence-Based Clinical Practice Guidelines* [23]; conversely no TPX prescription in high-risk patients and TPX prescription in low-risk patients was considered as inappropriate. Given that classification of high and low VTE risk is dependent on the particular RAM used, the results on over-, under-, appropriate, and inappropriate use varied, based on which RAM was considered. Finally, we assessed prescription of mechanical and pharmacologic TPX among high-risk and low-risk patients with a contraindication to pharmacologic TPX.

Finally, we also assessed clinical outcome events, including symptomatic VTE during 90 days after study inclusion, in-hospital clinically relevant bleeding, and major bleeding. Symptomatic VTE included objectively confirmed PE, distal and proximal DVT of the upper and lower extremity [20]. In-hospital clinically relevant bleeding was defined as combined major and clinically relevant non-major bleeding. The definition of major bleeding was based on the criteria from the *International Society of Thrombosis and Haemostasis*, which includes fatal bleeding and/or symptomatic bleeding in a critical area or organ (such as intracranial, intraspinal, intraocular, retroperitoneal, intra-articular, pericardial, or intramuscular with compartment syndrome) and/or bleeding with a reduction of hemoglobin ≥20 g/L, or leading to the transfusion ≥2 units of packed red blood cells [24]. Clinically relevant non-major bleeding referred to overt bleeding that does not meet criteria for major bleeding but is associated with a medical intervention, bleeding important enough to be documented in the medical chart for inpatients, or bleeding resulting in pain or impairment of activities of daily living [20]. VTE and bleeding outcomes were adjudicated by 3 independent clinical experts.

### 2.4 Statistical analysis

Patient characteristics were presented using descriptive statistics. We calculated the proportion of patients at high and at low VTE risk according to each RAM. In addition, we assessed the proportion of patients who would have been categorized as high risk and low risk by all 4 RAMs, respectively. The proportion of overall TPX at baseline and anytime during the entire hospitalization was calculated for high-risk and low-risk patients based on each score, and for patients categorized as high- or low-risk by all 4 RAMs, respectively. The proportion of VTE outcomes during 90 days, in-hospital clinically relevant bleeding, and major bleeding was presented for categories of underuse, appropriate use, and overuse of any TPX during hospitalization based on each RAM, and compared using the chi-squared test. All analyses were performed using Stata statistical software, Release 16 (Stata Corporation, College Station). Two-sided *P* values < .05 were considered statistically significant.

## 3 Results

**Figure 1:**
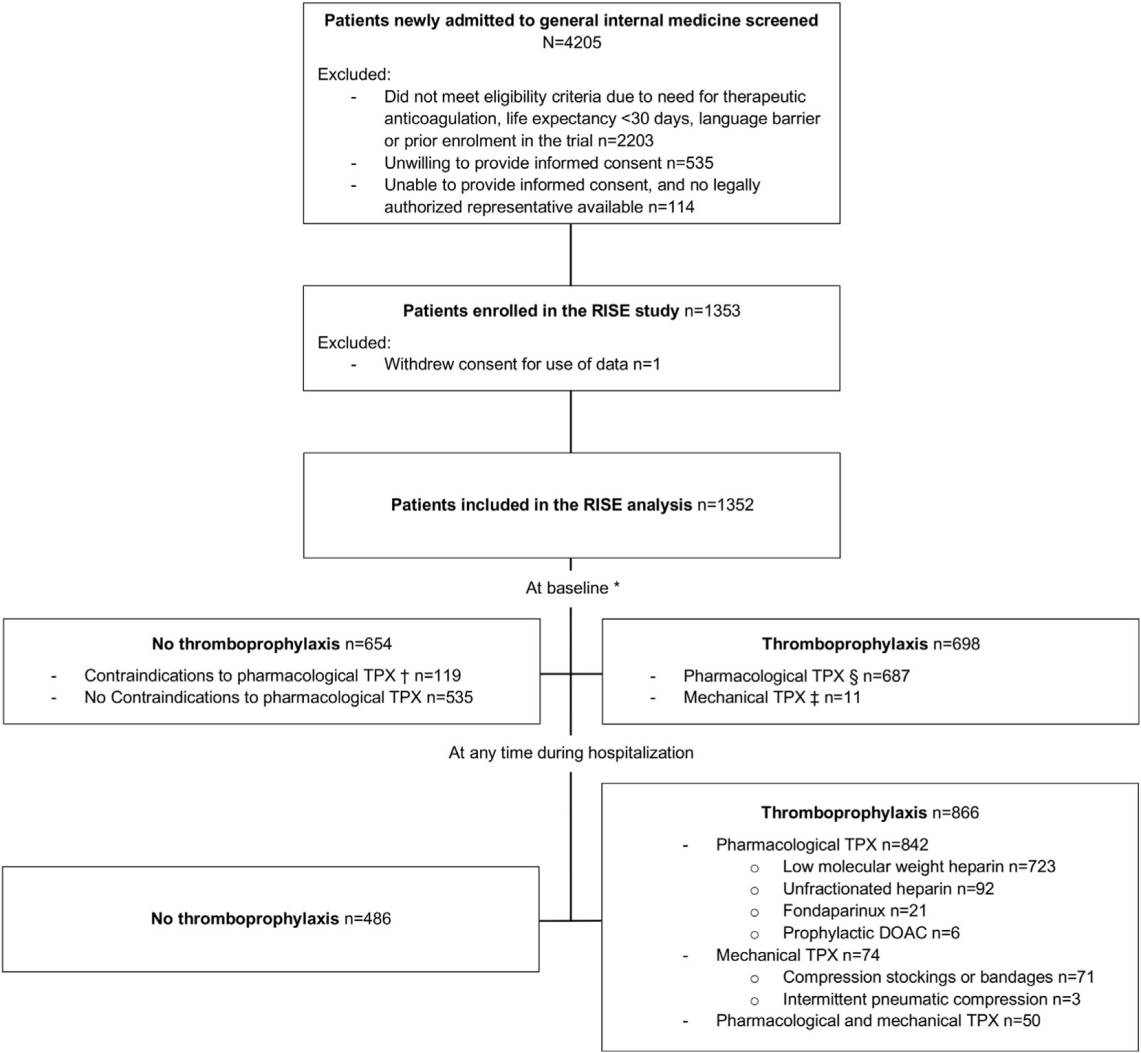
Prescription and type of thromboprophylaxis in medical inpatients at baseline and at any time during hospitalization for at least one day. ∗ Within 72 hours (median 24 hours) of admission. † Defined as liver failure or any other active bleeding disorder, active bleeding, or hemorrhagic transformation of acute ischemic stroke. § Defined as low-molecular-weight heparin, unfractionated heparin, fondaparinux, or direct oral anticoagulants in a prophylactic dose. ‡ Defined as use of lower extremity compression stockings or bandages, or intermittent pneumatic compression devices. TPX, thromboprophylaxis.

Overall, 1352 medical inpatients were included in the study (Figure 1). Among all participants, the median age was 67 years (IQR, 54-77 years), 590 (43.6%) were female **(**Table 2**)**, and the median duration of hospital stay was 6 days (IQR, 4-10 days). The most common risk factors for VTE were older age, acute infection, reduced mobility or immobilization for ≥3 days, obesity, and active cancer **(**Table 2**)**. Given that we enrolled only patients that were admitted for hospitalization to general internal medicine wards, none of the participants had a stay in intensive or coronary care unit at baseline.

**Table 2:** Characteristics of all participants included in the RISE analysis (*n* = 1352).

Characteristics	*n* (%)
**Baseline characteristics**	
Age, y, median (IQR)	67 (54-77)
Female sex	590 (43.6)
Body mass index in kg/m^2^, mean (SD)	25.8 (6.1)
**VTE risk factors**	
Previous VTE^a^	88 (6.5)
Hypercoagulable state or thrombophilia^b^	12 (0.9)
Active cancer^c^	263 (19.5)
Myeloproliferative syndrome^d^	12 (0.9)
Cardiac failure^e^	134 (9.9)
Respiratory failure^f^	237 (17.5)
Acute infection	581 (43.0)
Acute rheumatologic disorder	54 (4.0)
Reduced mobility for ≥3 d^g^	485 (35.9)
Immobilization for ≥3 d^h^	382 (28.3)
Immobilization for ≥7 d^i^	110 (8.1)
Paresis or paralysis of lower extremities	28 (2.1)
Age >60 y	846 (62.6)
Age ≥70 y	588 (43.5)
Obesity / BMI ≥30 kg/m^2^	269 (19.9)
Stroke (≤3 mo)	12 (0.9)
Stroke (≤1 mo)	9 (0.7)
Myocardial infarction (≤1 mo)	26 (1.9)
Nephrotic syndrome	7 (0.5)
Hormonal treatment^j^	58 (4.3)
Travel >6 h (≤7 d)	36 (2.7)
Chronic venous insufficiency	254 (18.8)
Pregnancy	4 (0.3)
Dehydration	158 (11.7)
Surgery (≤1 mo)	49 (3.6)
Trauma (≤1 mo)	84 (6.2)
Stay in intensive or coronary care unit	0 (0)
**Contraindications to pharmacologic TPX**	
Any contraindication for pharmacologic TPX^k^	119 (8.8)
Liver failure^l^	10 (0.7)
Any active bleeding	89 (6.6)
Hemorrhagic transformation or acute ischemic stroke	0 (0)
Any active bleeding disorder^m^	36 (2.7)

BMI, body mass index; TPX, thromboprophylaxis; VTE, venous thromboembolism.

a Defined as prior deep vein thrombosis or pulmonary embolism.

b Defined as antithrombin deficiency, activated protein C resistance, protein C or protein S deficiency, factor (F)V Leiden, G20210A prothrombin-mutation, or antiphospholipid syndrome.

c Defined as metastatic cancer, or cancer treated with radiotherapy, chemotherapy, immunotherapy, or cancer surgery within last 6 mo.

d Refers to essential thrombocytopenia, polycythemia vera, myelofibrosis, or chronic myeloid leukemia.

e Acute or chronic cardiac failure, defined as diagnosis of heart failure preserved or reduced or ejection fraction in medical records, or known left ventricular ejection fraction of <40%.

f Acute or chronic respiratory failure, defined as need for supplemental oxygen.

g Defined as anticipated bed rest with or without bathroom privileges for ≥3 d.

h Defined as (anticipated) complete bedrest or inability to walk for >30 min/d for ≥3 d.

i Defined as confinement to chair or bed with or without bathroom privileges for ≥7 d immediately prior to and (anticipated) during hospital admission.

j Refers to hormonal contraception, postmenopausal hormone therapy, antitumor therapy containing estrogen, ethinylestradione, or estradiol.

k Defined as liver failure, any other active bleeding disorder, active bleeding, or hemorrhagic transformation of acute ischemic stroke.

l Defined as diagnosis of liver failure in medical records, or cirrhosis with spontaneous international normalized ratio >2.

m Defined as the presence of any bleeding disorder except for liver disease, eg, hemophilia, von Willebrand disease, idiopathic thrombocytopenia.

### 3.1 Risk of VTE according to validated RAMs

**Figure 2:**
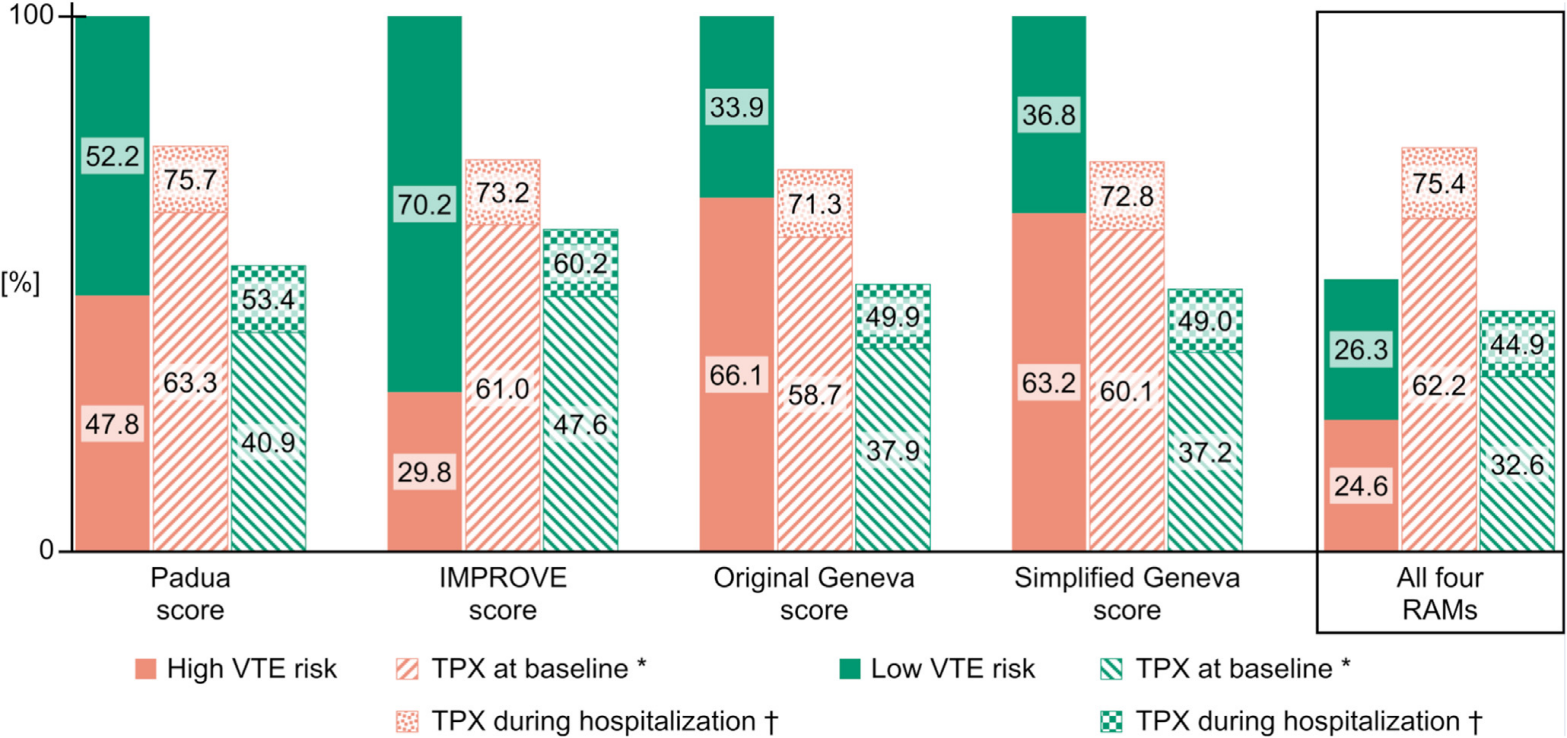
Proportion of medical inpatients at high and low venous thromboembolism risk according to validated RAMs and related prescription of TPX. Variables to calculate VTE risk according to each RAM were collected at baseline (ie, within 72 hours [median 24 hours] of admission). IMPROVE, *International Medical Prevention Registry on Venous Thromboembolism*; RAMs, risk assessment models; TPX, thromboprophylaxis; VTE, venous thromboembolism. ∗ Refers to prescription of mechanical or pharmacologic TPX at baseline. † Refers to prescription of mechanical or pharmacologic TPX anytime during the entire hospitalization for at least one day.

According to the Padua score, 646 (47.8%) patients were categorized as high risk. The IMPROVE score categorized 403 (29.8%) patients as high risk. Based on the original and simplified Geneva score, 893 (66.1%) and 854 (63.2%) patients were classified as high risk, respectively. Overall, 333 (24.6%) of patients were consistently categorized as high risk, and 356 (26.3%) as low risk by all 4 RAMs (Figure 2).

### 3.2 Overuse and underuse of TPX

At baseline, 698 (51.6%) patients had a prescription for any TPX (mechanical TPX *n* = 11, pharmacologic TPX *n* = 687). During the entire hospitalization, 866 (64.1%) patients had a prescription for any TPX. Of these, 842 patients were prescribed a pharmacologic TPX (type and dose shown in Supplementary Table S1) and 74 patients, a mechanical TPX (combined mechanical and pharmacologic TPX in 50 patients). The most common pharmacologic TPX prescribed was LMWH, followed by UFH (Figure 1). The median duration of pharmacologic TPX was 5 days (IQR, 3-8 days); TPX was started on the day of admission in 34.5% and until the first day after admission in 76.4% of patients receiving any TPX during hospitalization **(**Supplementary Table S2**)**. In most patients (*n* = 71), compression stockings or bandages were used for mechanical TPX (Figure 1).

Depending on the RAM used, 58.7% to 63.3% of high-risk patients had a prescription of any TPX at baseline. Throughout the hospital stay the proportion increased from 71.3% to 75.7% (Figure 2). Thus, the proportions of patients categorized as high risk who were not prescribed any TPX at baseline and during the entire hospitalization (ie, TPX underuse) were 36.7% to 41.3% and 24.3% to 28.7%, respectively. In contrast, 37.2% to 47.6% and 49.0% to 60.2% of patients categorized as low risk by any of the RAMs were prescribed any TPX at baseline and during the entire hospitalization (ie, TPX overuse), respectively (Figure 2). The results were similar in patients who were grouped in the same risk category by all 4 RAMs. Among patients consistently categorized as high risk by all 4 RAMs, 62.2% had a prescription of TPX at baseline and 75.4% at any time during hospitalization, while among patients consistently categorized as low risk it was 32.6% and 44.9%, respectively (Figure 2).

### 3.3 Patients with a contraindication to pharmacologic TPX

Overall, 119 (8.8%) of patients had at least one or several contraindications to pharmacologic TPX, including liver failure, any other active bleeding disorder, or active bleeding **(**Table 2**)**. Despite the presence of a contraindication, 26 patients were prescribed pharmacologic TPX at baseline. Among patients with a contraindication, 38 patients were consistently categorized as high risk and 41 consistently as low risk by all 4 RAMs. TPX was prescribed to 14 high-risk patients with a contraindication (pharmacologic TPX only in 12 patients) and to 8 low-risk patients with a contraindication (all with pharmacologic TPX only; Table 3).

**Table 3:** Prescription of TPX in medical inpatients with a contraindication to pharmacologic TPX at baseline.

Patients with a contraindication to pharmacologic TPX	Overall^a^	Any TPX^b^	Pharmacologic TPX^c^	Mechanical TPX^d^
	*n* (%)	N		
Overall	119 (8.8)	29	26	3
High VTE risk according to all 4 RAMs	38 (2.8)	14	12	2
Low VTE risk according to all 4 RAMs	41 (3.0)	8	8	0

RAM, risk assessment model; TPX, thromboprophylaxis; VTE, venous thromboembolism.

Variables to calculate VTE risk according to each RAM and information on TPX were collected at baseline (ie, within 72 hours [median 24 hours] of admission). Contraindications to pharmacologic TPX include liver failure, or any other active bleeding disorder, active bleeding, or hemorrhagic transformation of acute ischemic stroke.

a The proportion refers to the overall RISE study population (*N* = 1352).

b Relates to prescription of any mechanical or pharmacologic TPX.

c Defined as low-molecular-weight heparin, unfractionated heparin, fondaparinux, or direct oral anticoagulants in a prophylactic dose.

d Defined as prescription of lower extremity compression stockings or bandages, or intermittent pneumatic compression devices.

### 3.4 Venous thromboembolism and bleeding outcomes according to underuse, appropriate use, and overuse of TPX

A total of 28 (2.1%) VTE events occurred during 90 days after study inclusion. There were no significant differences in VTE outcomes between groups with underuse, appropriate use, or overuse of TPX, irrespective of the RAM used **(**Table 4**)**. During their hospital stay, 64 (4.7%) patients suffered from a clinically relevant bleeding event, and 34 (2.5%) had major bleeding. Overall, risk for both in-hospital clinically relevant bleeding as well as in-hospital major bleeding tended to be increased in high VTE risk patients with underuse of TPX, and lower in patients at low VTE risk patients with overuse of TPX compared to patients with appropriate TPX prescription. However, the difference was only statistically significant for in-hospital clinically relevant bleeding in groups of underuse, appropriate use, or overuse of TPX based on the IMPROVE score **(**Table 4**)**. Results for bleeding risk were similar after exclusion of 40 participants who were started on therapeutic dose anticoagulation during the index hospitalization **(**Supplementary Table S3**)**.

**Table 4:** VTE within 90 days and in-hospital bleeding events according to appropriateness of TPX use based on each RAM.

RAM	Underuse of TPX^a^	Appropriate use of TPX^b^	Overuse of TPX^c^	*P* value
	**VTE events within 90 d / n participants (%)**			
Padua score	4/157 (2.6)	16/818 (2.0)	8/377 (2.1)	.89
IMPROVE score	2/108 (1.9)	13/673 (1.9)	13/571 (2.3)	.90
Simplified Geneva score	3/232 (1.3)	22/876 (2.5)	3/244 (1.2)	.30
Original Geneva score	3/256 (1.2)	23/867 (2.7)	2/229 (0.9)	.13
High risk with all 4 RAMs	2/82 (2.4)	7/251 (2.8)	-	.87
Low risk with all 4 RAMs	-	1/196 (0.5)	1/160 (0.6)	.89
	**In-hospital clinically relevant bleeding events / n participants (%)**			
Padua score	12/157 (7.6)	38/818 (4.7)	14/377 (3.7)	.15
IMPROVE score	10/108 (9.3)	37/673 (5.5)	17/571 (3.0)	.008
Simplified Geneva score	15/232 (6.5)	44/876 (5.0)	5/244 (2.1)	.06
Original Geneva score	14/256 (5.5)	44/867 (5.1)	6/229 (2.6)	.25
High risk with all 4 RAMs	9/82 (11.0)	18/251 (7.2)	-	.27
Low risk with all 4 RAMs	-	9/196 (4.6)	4/160 (2.5)	.30
	**In-hospital major bleeding events / n participants (%)**			
Padua score	5/157 (3.2)	21/818 (2.6)	8/377 (2.1)	.77
IMPROVE score	5/108 (4.6)	19/673 (2.8)	10/571 (1.8)	.17
Simplified Geneva score	7/232 (3.0)	26/876 (3.0)	1/244 (0.4)	.07
Original Geneva score	7/256 (2.7)	24/867 (2.8)	3/229 (1.3)	.44
High risk with all 4 RAMs	4/82 (4.9)	10/251 (4.0)	-	.73
Low risk with all 4 RAMs	-	5/196 (2.6)	1/160 (0.6)	.16

RAM, risk assessment model; TPX, thromboprophylaxis; VTE, venous thromboembolism.

a Refers to failure to prescribe any TPX during hospitalization to patients categorized as high VTE risk.

b Refers to prescription of any TPX during hospitalization in high-risk patients and no TPX prescription in low-risk patients.

c Refers to prescription of any TPX during hospitalization to patients categorized as low VTE risk.

## 4 Discussion

Our prospective multicenter cohort study showed that the proportion of medical inpatients categorized as high risk of VTE varies widely according to different validated RAMs. Only a quarter of patients were consistently categorized in the high risk group by all 4 RAMs. Overall, TPX at baseline was underused in up to 41% of high-risk and overused in up to 48% of low-risk patients. Overuse and underuse of TPX based on RAMs did not seem to be associated with adverse VTE and bleeding outcomes in our cohort, with similar VTE risk in patients with underuse, appropriate use or overuse of TPX.

Only half of patients were consistently categorized in the same risk group by all 4 RAMs. The proportion of patients classified as high risk varied widely from 30% to 66% according to different validated scores. Such large differences have also been shown in other studies [18,[25], [26], [27]]. For example, a recently published meta-analysis compared the Padua, the original Geneva, and the Caprini score and the American College of Chest Physicians criteria for VTE risk stratification, and found that 30% to 63% of patients were classified as high risk depending on the risk score used [25]. Although RAMs consist of some similar items, the wide variation in their estimation of which individuals are at high risk is due to variation in content and number of items, and possibly due to the fact that these items, eg, mobility, are defined and weighted differently. Current guidelines recommend to perform VTE risk stratification in medical inpatients to support clinical-decision making for TPX provision, but they acknowledge the uncertainty about optimal VTE risk stratification [10]. In a post hoc analysis of a prospective cohort study [18] and various systematic reviews [26,28,29], different RAMs have been compared in terms of their validity, applicability, and predictive accuracy. All RAMs have methodological and practical limitations, such as suboptimal sensitivity to identify high-risk patients [18,26], non-uniform cut-off values to define low and high risk groups [14], or excessive complexity [17], that could limit their use in clinical practice [26].

Our study showed that only about two-thirds of patients classified as high risk had an appropriate prescription of any TPX at baseline, while this increased up to 75% when considering prescription of any TPX during the entire hospitalization, resulting in an estimate of TPX underuse of 25% to 30% in high-risk patients. The issue of underuse of TPX in high-risk patients is well known. In the multinational cross-sectional Epidemiologic International Day for the Evaluation of Patients at Risk for Venous Thromboembolism in the Acute Hospital Care Setting (ENDORSE) study including approximately 38,000 medical inpatients from 32 countries, around 40% were categorized as high VTE risk by the American College of Chest Physicians criteria. TPX underuse was observed in up to 60% of high-risk patients [12]. In a recently published systematic review and meta-analysis of studies that included 135,000 medical inpatients from 20 countries, only about 55% of high-risk patients had a prescription of pharmacologic TPX [25]. A potential explanation for the higher estimates of TPX underuse in these studies compared to ours is that appropriateness of TPX prescription may differ according to geographic regions [25]. TPX is influenced by many factors, such as national guidelines, physician and public VTE awareness, health system standards, or reimbursement [25,30,31]. In addition, a physician’s estimation regarding an increased bleeding risk could contribute to underuse of TPX in selected patients at high VTE risk. This potential explanation for TPX underuse is supported by our results showing a trend toward an increased risk of in-hospital bleeding events in patients with underuse of TPX. Considering only the results for Switzerland in the ENDORSE study (61%) and for Europe in the meta-analysis (67%), the percentage of high-risk patients with an appropriate TPX prescription is similar to our findings. As a result of underuse, high-risk patients may be unnecessarily exposed to VTE risk. However, we did not find an increased risk of VTE events in patients with underuse of TPX in our study; in fact, VTE risk was similar in groups of underuse, appropriate use, and overuse of TPX. Given that the incidence of hospital-acquired VTE in medical patients can be decreased by >50% with appropriate TPX based on historical randomized controlled trials [7,8], this observation suggests that current RAMs may be suboptimal to predict VTE risk [26], or the current real-life impact of TPX is overestimated.

Overuse of TPX seems to be even more pronounced than its underuse. This results in a substantial proportion of low-risk patients that are unnecessarily exposed to an increased risk of bleeding (approximately 1.6-fold increased risk of major bleeding with any heparin, with a lower risk in patients receiving LMWH compared with UFH) [8], heparin-induced thrombocytopenia, as well as potentially painful TPX injections. However, based on our results, patients at low VTE risk in whom TPX is overused in our cohort seem to be those at particular low risk of bleeding. Unlike with underuse, the percentage of overuse was more dependent on the RAM used. The proportion of overuse was smallest with the use of both Geneva scores, which is not surprising, as these 2 RAMs classify more patients as high risk compared with other RAMs [18]. In previous studies, estimates of overuse were somewhat smaller or comparable to our study. In the ENDORSE study, 30% of low-risk patients were prescribed any TPX [12]. In another study, the overuse of TPX in low-risk patients was around 48% to 57% [17,26]. A potential explanation for overuse of TPX could be the concern about patient safety, as the risks of unnecessary TPX may be outweighed by the risk of a VTE event that could potentially be prevented [32]. A previous study could not identify any clinical factors predicting the overuse of TPX in low risk patients, and the authors hypothesized that non-clinical factors such as local habits may play a role [33].

About 3% of all VTE high-risk patients had a contraindication to pharmacologic TPX. Nonetheless, approximately one-third of these patients were prescribed pharmacologic TPX, which is consistent with findings of a previous study [34]. Despite guidelines recommending to prescribe mechanical TPX among high-risk patient with a contraindication to pharmacologic TPX [3,10], mechanical TPX was only prescribed in a minority of these patients in our study, suggesting that physicians seem to be insufficiently aware of this option or participating hospitals do not follow this recommendation. Another possible explanation could be the limited evidence for benefit of mechanical prophylaxis in medical inpatients [10], with a concern for harm, such as skin damage on the legs due to intermittent pneumatic compression [35]. Among low-risk patients with a contraindication, one-quarter was prescribed pharmacologic TPX. Even though the absolute number of patients was small, this result is alarming given that they were unnecessarily exposed to an increased bleeding risk associated with TPX.

Evidence of underuse and overuse emphasizes the need for increased VTE awareness to optimize VTE prevention [30]. VTE awareness campaigns, such as the annual World Thrombosis Day that has been launched in 2014, have a growing but still insufficient impact [36]. The American Heart Association and the International Society of Thrombosis and Haemostasis have drafted a scientific statement outlining their implementation in practice to improve VTE prevention [37]. Besides the lack of awareness, another potential explanation for inappropriate TPX use is the uncertainty about optimal VTE risk stratification of medical inpatients by physicians, which may arise from the lack of an optimal and easy-to-use RAM and the inconsistent classification of patients into VTE risk groups by various existing RAMs, as shown in our study [38]. Consequently, RAMs do not seem to be consistently used in clinical practice to guide TPX prescription. A prospective cohort study with dedicated collection of RAM items allowing a head-to-head comparison of validated RAMs in hospitalized medical inpatients is currently lacking and needed to provide clear guidance for physicians about optimal VTE risk assessment. In addition, objectively measurable items could potentially help to standardize risk stratification and ultimately classification into risk groups. However, even an ideal and standardized risk assessment strategy will only improve appropriateness of TPX if it is applied correctly in everyday clinical practice. The introduction of institutional guidelines does not seem to sufficiently improve adequacy of TPX prescription, as shown previously [39]. Computer-alert programs with the integration of a RAM to identify high-risk patients as well as contraindications to pharmacologic TPX may improve TPX prescription and decrease in the rate of VTE compared to usual care [40]. However, evidence on the beneficial effect of computer alert systems are inconsistent, as electronic alerts may be ignored by physicians [41].

To our knowledge, this is the first multicenter, prospective cohort study with dedicated collection of RAM items and assessment of different validated RAMs and TPX use in newly admitted medical inpatients. There are several previous studies which applied different RAMs on the same population to access their external validity [42,43]. However, our study is the first to examine how many of the patients were consistently classified as high risk or low risk using all RAMs, thus, showing how the individual RAMs differ in classifying a particular patient. However, several limitations should be noted. The generalizability of the study results may be limited to a tertiary care hospital setting of high-income countries with comprehensive healthcare insurance and mainly Caucasian population, given that it was performed in Swiss university hospitals only, and detailed information on race and ethnicity was not collected. In addition, we cannot rule out that physicians changed their TPX prescription habit due to the conduct of this study. However, we communicated that the study investigated mobility in hospitalized medical inpatients (a secondary goal of the RISE cohort [20]), but did not inform physicians explicitly about the aim to investigate VTE prevention strategies and outcomes, which was also the reason why we were not able to compare the performance RAMs to subjective clinical gestalt. Another potential limitation of the study is that the appropriateness of TPX prescription is only based on VTE RAMs without including a bleeding RAM. As suggested by our results showing no difference in VTE risk among those with underuse of TPX but a trend toward a higher risk of bleeding than those with appropriate TPX use, clinicians may be making tradeoffs between thrombosis and bleeding risk when considering pharmacologic TPX in medical inpatients, which may have contributed in part to the underuse of pharmacological TPX.

## 5 Conclusions

Our study demonstrated that the risk stratification of VTE varies widely across validated RAMs. Only half of the patients were consistently classified into the same risk group by all 4 RAMs. While TPX remains underused in high-risk patients, overuse in low-risk patients is even more pronounced. However, we did not find a negative impact of inappropriate TPX on VTE and bleeding outcomes, which may suggest suboptimal performance of current RAMs. In addition, underuse of TPX in some patients classified as high VTE risk may have been appropriate based on clinicians’ concerns for bleeding risk. Further studies are needed to identify optimal risk assessment strategies to improve VTE prevention in hospitalized medical inpatients.
