# Supplementary material for: Overuse and underuse of thromboprophylaxis in medical inpatients
Source: Res Pract Thromb Haemost. 2023 Aug 23;7(6):102184. doi: 10.1016/j.rpth.2023.102184 (PMC10514554; doi:10.1016/j.rpth.2023.102184)
Supplement: Supplementary material [file mmc1.docx]

**Supplementary Material**

**Supplemental Table 1.** Doses and types of thromboprophylactic agents used in the RISE study.

| **Thromboprophylactic agents** | **Doses** | **Patients, n** |
| --- | --- | --- |
| Enoxaparin | up to 40mg/day | 723 |
| Fondaparinux | 2.5mg 1x/day | 21 |
| Unfractionated heparin | up to 15’000 IU/day | 92 |
| Rivaroxaban | 10mg 1x/day | 6 |
| Apixaban | 2.5mg 2x/day in the absence of an indication for therapeutic anticoagulation (e.g. atrial fibrillation) |  |

**Supplemental Table 2.** Timing of thromboprophyaxis (TPX) among participants receiving any pharmacological TPX during hospitalization (n=842).

| **Timing of TPX** | **n (%)** |
| --- | --- |
| Duration of TPX in days, median (IQR) | 5 (3-8) |
| TPX started on the day of admission | 299 (34.5) |
| TPX started until day 1 after admission | 662 (76.4) |
| TPX started until day 2 after admission | 765 (88.3) |

Abbreviations: IQR, interquartile range; TPX, thromboprophylaxis

**Supplemental Table 3.** In-hospital bleeding events according to appropriateness of TPX use based on each RAM, after exclusion of participants who were started on therapeutic dose anticoagulation during hospitalization (n=40).

| **RAM** | **Underuse of TPX** * | **Appropriate use of TPX** † | **Overuse of TPX** ‡ | **p-value** |
| --- | --- | --- | --- | --- |
|  | **in-hospital clinically relevant bleeding events / n participants (%)** | | |  |
| Padua score | 12/152 (7.9) | 35/793 (4.4) | 12/367 (3.3) | 0.07 |
| IMPROVE score | 10/103 (9.7) | 33/654 (5.1) | 16/555 (2.9) | 0.006 |
| Simplified Geneva score | 15/227 (6.6) | 39/847 (4.6) | 5/238 (2.1) | 0.06 |
| Original Geneva score | 14/252 (5.6) | 39/838 (4.7) | 6/222 (2.7) | 0.31 |
| High risk with all four RAMs | 9/79 (11.4) | 15/238 (6.3) | - | 0.14 |
| Low risk with all four RAMs | - | 9/193 (4.7) | 4/156 (2.6) | 0.30 |
|  | **in-hospital major bleeding events /**  **n participants (%)** | | |  |
| Padua score | 5/152 (3.3) | 18/793 (2.3) | 6/367 (1.6) | 0.50 |
| IMPROVE score | 5/103 (4.9) | 15/654 (2.3) | 9/555 (1.6) | 0.12 |
| Simplified Geneva score | 7/227 (3.1) | 21/847 (2.5) | 1/238 (0.4) | 0.10 |
| Original Geneva score | 7/252 (2.8) | 19/838 (2.3) | 3/222 (1.4) | 0.56 |
| High risk with all four RAMs | 4/79 (5.1) | 7/238 (2.9) | - | 0.37 |
| Low risk with all four RAMs | - | 5/193 (2.6) | 1/156 (0.6) | 0.16 |

Abbreviations: RAM, risk assessment model; TPX, thromboprophylaxis

* refers to failure to prescribe any TPX during hospitalization to patients categorized as high VTE risk

† refers to prescription of any TPX during hospitalization in high-risk patients and no TPX prescription in low-risk patients

‡ refers to prescription of any TPX during hospitalization to patients categorized as low VTE risk
